# The pulmonary blood volume variation is higher in patients with heart failure compared to healthy controls

**DOI:** 10.1186/1532-429X-16-S1-P288

**Published:** 2014-01-16

**Authors:** Mikael Kanski, Martin Ugander, Rasmus Borgquist, Håkan Arheden

**Affiliations:** 1 Department of Clinical Physiology, Lund University and Lund University Hospital, Lund, Lund, Sweden; 2 Department of Clinical Physiology, Karolinska Institutet and Karolinska University Hospital, Stockholm, Sweden; 3 Department of Cardiology, Lund University and Lund University Hospital, Lund, Lund, Sweden

## Background

In left ventricular heart failure, blood will congest backwards to the pulmonary circulation and result in an increased pulmonary vascular resistance. If the increase in pressure result in impaired gas exchange due to pulmonary oedema, the pulmonary blood will redistribute to better ventilated areas in order to maintain adequate gas exchange[1]. This might affect the pulsatility of the pulmonary blood flow which can be quantified by measuring the pulmonary blood volume variation (PBVV)[2]. Therefore, the aim of this study was to investigate if PBVV in patients with heart failure differed from healthy controls.

## Methods

: Fifteen patients with heart failure and 10 healthy volunteers underwent cardiac MRI at 1.5T. Flow measurements were acquired in the pulmonary trunk and all pulmonary veins. The PBVV was calculated by integrating the flow in all vessels leading to and from the pulmonary circulation. The PBVV was indexed to right ventricular stroke volume (PBVV/SV). Patients underwent a 6-minute walk test (6MWT) as an independent measure of functional capacity.

## Results

**Figure 1:**
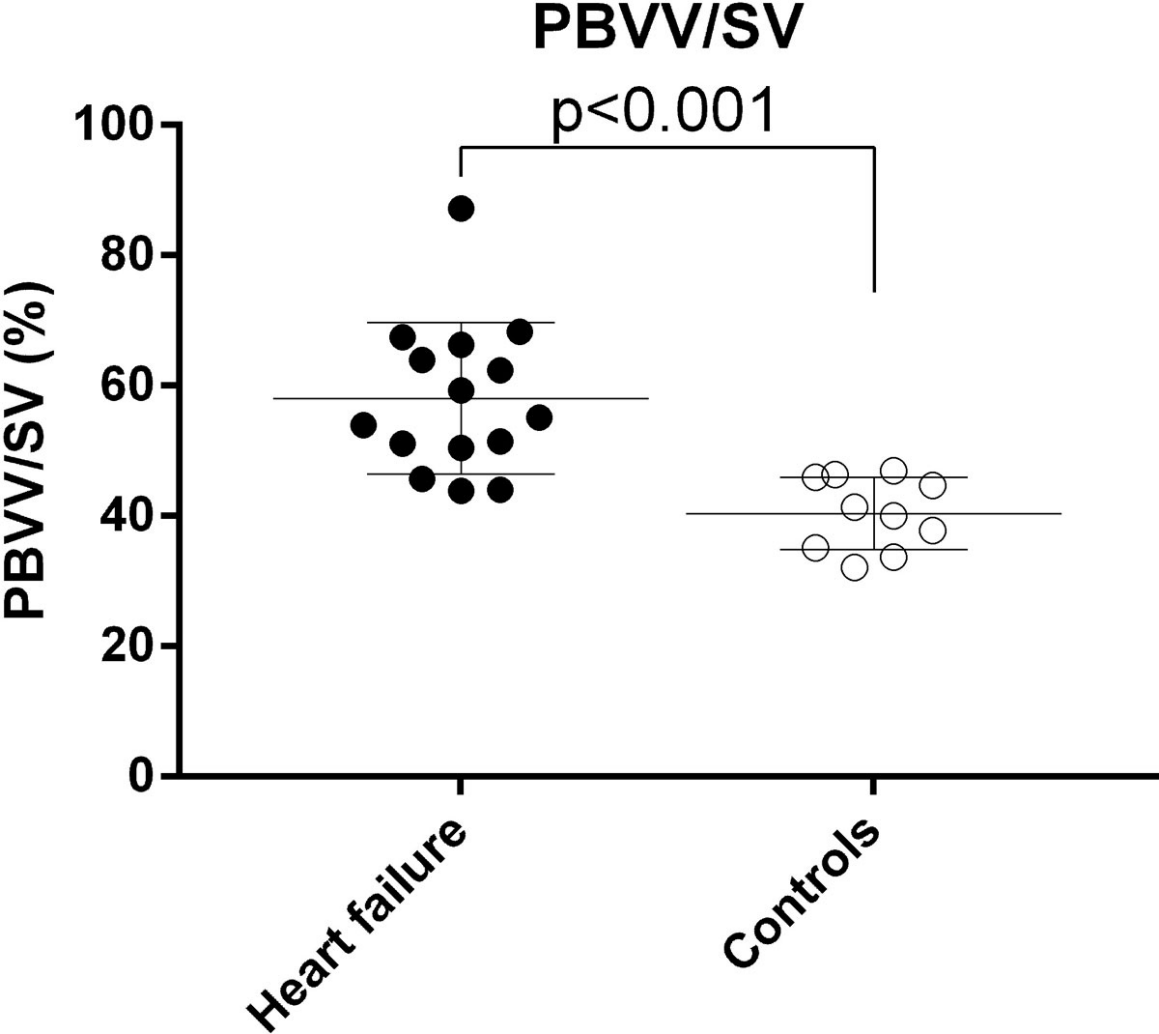
**The pulmonary blood volume variation (PBVV) indexed to stroke volume (SV) in patients with heart failure (black circles) and healthy controls (open circles)**. Error bars show mean ± SD. Note the statistically significant difference between patients and controls in PBVV/SV (%).

Left ventricular ejection fraction (LVEF) in patients with heart failure and healthy controls were 27 ± 6 and 60 ± 3%, respectively (p < 0.001). Patients had a significantly higher PBVV/SV compared to controls (58 ± 12% vs 40 ± 5%, p < 0.001, Figure 1), 3/15 (20%) patients had PBVV/SV values within the range of healthy controls. In patients, there was no relationship between PBVV/SV and the LVEF (r2 = 0.13, p = 0.18), atrioventricular plane displacement (AVPD, r2 = 0.17, p = 0.12), or 6-minutes walk test (6MWT, n = 8, r2 = 0.00, p = 0.90).

## Conclusions

Patients with heart failure had a higher PBVV/SV compared to healthy controls. This can be explained by a pulmonary circulatory response, either active or passive, to the failing left ventricle. However, the lack of correlation between PBVV/SV and LVEF, AVPD, or 6MWT suggests an active process in order to maintain an adequate gas exchange in the lungs. Therefore, we propose that PBVV/SV may be a measure of the pulmonary circulatory involvement caused by heart failure.

## Funding

Swedish Research Council, Swedish Heart and Lung Foundation, Medical Faculty at Lund University, Region of Scania.
